# Effect of a Biosurfactant Extract Obtained from a Corn Kernel Fermented Stream on the Sensory Colour Properties of Apple and Orange Juices

**DOI:** 10.3390/foods12101959

**Published:** 2023-05-11

**Authors:** Benita Pérez-Cid, Susana Río Segade, Xanel Vecino, Ana Belén Moldes, José Manuel Cruz

**Affiliations:** 1 Analytical and Food Chemistry Department, Faculty of Chemistry-CINTECX, University of Vigo, Campus As Lagoas-Marcosende, 36310 Vigo, Spain; benita@uvigo.gal; 2 Dipartimento di Scienze Agrarie, Forestali e Alimentari, Università degli Studi di Torino, Largo Paolo Braccini 2, 10095 Turin, Italy; susana.riosegade@unito.it; 3 Chemical Engineering Department, School of Industrial Engineering-CINTECX, University of Vigo, Campus As Lagoas-Marcosende, 36310 Vigo, Spain; amoldes@uvigo.gal (A.B.M.); jmcruz@uvigo.gal (J.M.C.)

**Keywords:** colour parameters, CIELAB system, orange juice, apple juice, corn kernel extract

## Abstract

In this work, we assessed the effect of a biosurfactant extract, which possesses preservative properties, on the sensory properties, regarding colour, of two fruit juices: pasteurized apple juice and natural orange juice. This biosurfactant extract was obtained from corn steep liquor, a secondary stream of the corn wet-milling industry. The biosurfactant extract is composed of natural polymers and biocompounds released during the spontaneous fermentation of corn kernels during the steeping process of corn. The reason for this study is based on the importance of colour as a visual attribute that can determine the consumer’s preferences; it is important to study the effect of the biosurfactant extract under evaluation before including it in juice matrices. For this, a surface response factorial design was employed and the effects of the biosurfactant extract concentration (0–1 g/L), the storage time (1–7 days), and the conservation temperature (4–36 °C) on the CIELAB colour parameters (L*, a*, b*) of the juice matrices were evaluated, as well as the total colour differences (ΔE*) regarding the control juices and the saturation index ($C_{\mathrm{ab}}^{*}$). Moreover, the CIELAB coordinates of each conducted treatment were converted into RGB values to obtain visual colour differences that can be appreciated by testers or consumers.

## 1. Introduction

Colour is one of the most important visual attributes of fresh vegetables and fruits; it is directly associated to the concept of quality that strongly influences the consumers’ acceptability of a product. In particular, the visual assessment of fruit juices could also encourage other sensory parameters such as flavour, sourness, and freshness [1] and affects the overall perceived quality and even the purchase decision making. In fact, the study of colour characteristics is attracting growing attention from the juice industry due to the possible economic repercussions. For each fruit, the juice colour depends on each specific variety, environmental growing conditions and other agronomic factors. However, it is well known that fruit processing and juice storage could also have a strong effect on colour changes and other quality attributes [2,3]. Therefore, different thermal or non-thermal treatments such as high-pressure processes [4,5,6,7] have been widely evaluated to inactivate the microbial proliferation and enzymatic reactions of juices, which negatively affect the fresh quality properties of juices matrices such as colour, flavour, aroma, vitamin content, texture, etc. In this way, the potential addition of biosurfactants to these food matrices as stabilizing agents could be highlighted, since it was proved that they possess antimicrobial and antiadhesive activities [8,9,10]. In fact, a biosurfactant extract obtained from a residual stream of the corn wet-milling industry showed a strong antimicrobial activity against some pathogenic bacteria including *Staphylococcus aureus, Escherichia coli*, and *Pseudomonas aeruginosa* [11]. The advantage of this biosurfactant extract in functional foods is based on its prebiotic character that has been demonstrated in a previous work; at the same time, the biosurfactant extract can prevent the growth of pathogenic microorganisms reducing the use of non-natural preservative agents [12]. Despite these properties and considering the microbial origin of the biosurfactants, nowadays its use is very limited in the food industry and almost no research works were found on it. In a previous work [13], the effect of a biosurfactant extract from corn steep liquor (CSL) in the maceration of red wines was proved, and it was observed that the presence of the biosurfactant promotes the extraction of anthocyanins from the skins during the maceration stage, which could be a promising alternative to preserve the colour properties of young red wines, thus opening an interesting potential for biosurfactants in the winemaking processes. In a recent work [14], the influence of a CSL biosurfactant extract on the stability and shelf-life of two fruit juices (pasteurized apple and natural orange) was also evaluated. It was observed that the presence of the biosurfactant mainly affected the pasteurized apple juice by considerably reducing the concentration of soluble sugars and reducing the glycemic index of the juice; these with negligible changes in the pH values, which is a sign of microbial stability. In this work it was also corroborated that the presence of the biosurfactant extract almost did not modify the biomass growth in both juice matrices studied.

On the other hand, it is important to indicate that colour attributes in foods can be evaluated by both instrumental and visual analysis [15], although it is well known that humans and instruments perceive colours in different ways. Whereas human colour perception is based on the responses of photoreceptors in the eye retina, instruments are capable of assessing the colour parameters in colorimetric coordinates such as those employed in the CIELAB or CIE 1976 L* a* b* colour space [16], this being one of the methodologies most widely used by producers and consumers for colour measurements in foods [17,18], including juice matrices [3,19]. This colour space, usually named the CIELAB system, correlates numerical colour values with human visual perception and provides more uniform colour differences in relation to the human receptors.

Taking the above into account, this work was focused on evaluating the colour changes caused by the addition of a CSL biosurfactant extract in two fruit juices (pasteurized apple and natural orange) with the aim to evaluate the effect of the biosurfactant extract on the colour attributes of both juice matrices. A surface response factorial design was applied to optimize the best experimental conditions to keep the visual properties of the juices, considering as independent variables the biosurfactant concentration and the storage time and temperature; the dependent variables were the CIELAB colour parameters (L*, a*, b*), together with the total colour differences (ΔE*) in respect to a control matrix juice, and the saturation index ($C_{\mathrm{ab}}^{*}$).

## 2. Materials and Methods

### 2.1. Biosurfactant Extract and Juice Samples

The natural orange juice (Hacendado Fresh brand) and the pasteurized apple juice (Hacendado brand) analysed in this work were acquired from the Mercadona supermarket (Spain). Before using in the different trials, both juices were centrifuged (Hettich, Rotina 380) at 5000 rpm for 30 min at 4 °C to remove the remaining pulp. On the other hand, the biosurfactant extract was obtained from CSL (Companhia Portuguesa de Amidos, S.A. San João da Talha, Portugal), by means of a liquid–liquid extraction with ethyl acetate following the experimental procedure previously described [20]. The biosurfactant extract under evaluation can decrease the surface tension of water up to 39.5 mN/m and possesses a critical micellar concentration (CMC) of 441 mg/L [14].

Colour parameters of the juice samples in the presence or absence of the biosurfactant extract were measured by means of an UV–Visible Spectrophotometer (Jasco V-650) equipped with a colorimeter that uses the CIELAB system and quartz cuvettes with a 10 mm optical pathway. The colour parameters of the two studied fruit juices, once centrifuged, were also measured before being included in the experiments of the factorial design and they are considered as control juices. All measurements for each sample were made in triplicate. L^*^ values represent the lightness of colour and range from 0 (black) to 100 (white), a* values represent greenness (−) to redness (+) coordinates, and b* values represent blueness (−) to yellowness (+) coordinates. Other parameters such as ΔE* and $C_{\mathrm{ab}}^{*}$ were also calculated by means of Equations (1) and (2) [3]:

(1) $$∆E^{*}=\sqrt{∆L^{*2}+∆a^{*2}+∆b^{*2}}$$

(2) $$C_{\mathrm{ab}}^{*}=\sqrt{{\;a}^{*2}+{\;b}^{*2}}\;$$

ΔE* is defined as the total colour difference between the control and treated samples, where ΔL* = L* − $L_{o}^{*}$; Δa* = a* − $a_{o}^{*}$; Δb* = b* − $b_{o}^{*}$; and the subscript letter ‘‘o’’ indicates the initial colour. The chroma ($C_{\mathrm{ab}}^{*}$) or saturation index determines the degree of difference of a hue in comparison with a grey colour with the same lightness and is considered a quantitative attribute of colourfulness.

### 2.2. Box–Behnken Factorial Design for Evaluating the Role of a Biosurfactant Extract on the Colour Properties of Fruit Juices

A Box–Behnken factorial design [21] was employed to evaluate the influence of a CSL biosurfactant extract on the colour properties of natural orange juice and pasteurized apple juice. For both juice matrices the independent variables optimized were the biosurfactant concentration (x_1_), storage time (x_2_), and temperature (x_3_), whereas the dependent variables were CIEL*a*b* colour parameters together with the calculated variables ΔE* and $C_{\mathrm{ab}}^{*}$. In Table 1 are shown the independent and dependent variables evaluated as well as the mathematical equations to code the independent variables in the range of study. 

In Table 2 can be observed the matrix experimental of the Box–Behnken factorial design, where the experiments were conducted in duplicate as follows: 25 mL of each juice (pasteurized apple juice or natural orange juice) was added to a plastic Falcon tube (50 mL) in presence of different concentrations of biosurfactant extract (0–1 g/L); temperature (4–36 °C); and storage time (1–7 days). Once finishing each experiment, the juices were centrifuged at 500 rpm for 30 min at 4 °C and the liquid phase was filtered through 0.45 µm polytetrafluoroethylene (PTFE) syringe filters. One aliquot of the filtered sample was used to measure the colour parameters (CIEL*, a* b* parameters) and the absorbance values at 420 nm.

### 2.3. Statistical Analysis

The Design-Expert Version software for Windows (Stat-Ease, Inc., Minneapolis, MN, USA) was employed to statistically analyse the experimental results obtained from the trials of the Box–Behnken factorial design. The treatment of the results provides theoretical equations that allowed prediction of the effect of the biosurfactant extract on the colour parameters of the two juice matrices studied. These equations were fitted to a second-order polynomial, as shown in Equation (3).

y = β_0_ + β_1_x_1_ + β_2_x_2_ + β_3_x_3_ + β_12_x_1_x_2_ + β_13_x_1_x_3_ + β_23_x_2_x_3_ + β_11_x_21_ + β_22_x_22_ + β_33_x_23 _ (3)

where y is the dependent variable, x represents the independent variables, and β indicates the regression coefficients obtained by the least squares method.

## 3. Results and Discussion

### 3.1. Colour Attributes of Fruit Juices from the Box–Behnken Factorial Design

The Box–Behnken factorial design was employed to evaluate the effect of a CSL biosurfactant extract on the sensory quality of two fruit juices (pasteurized apple and natural orange) under different operational conditions. It is important to indicate that the CSL biosurfactant extract employed in this work can be defined as an extract containing lipopeptides spontaneously produced by a *Bacillus* microorganism. It is mainly composed of C16-C18 fatty acids together with a mixture of amino acids (glutamine, asparagine, glycine, alanine, arginine, proline, and leucine) [22,23,24] as well as other bioactive compounds such as antioxidants or phospholipids [23]. We evaluated the ionic behaviour of the biosurfactant extract, observing its amphoteric properties due to the charges of the amino acids found in its composition [25]. The dependent variables quantified were the CIELAB colour parameters (L*, a*, b*), as well as the colour differences regarding the control samples (ΔE*) and the saturation index ($C_{\mathrm{ab}}^{*}$) defined as y_1_, y_2_, y_3_, y_4_, and y_5_. Table 2 shows the experimental results corresponding to all the dependent variables (y_1_–y_5_) studied in the 15 trials that constituted the experimental matrix of the factorial design applied in this work. In addition, in Table 3 can be observed the regression coefficients (β values) of each dependent variable and their statistical significance (*p*-values), together with the correlation coefficients (r^2^) of the mathematical fit. These coefficients are used to obtain theoretical equations that allow prediction of the behaviour of the dependent variables studied within the range established for each dependent variable in the factorial design (Table 1). These equations were established by considering the independent term (β_o_) and only the regression coefficients that were statistically significant (*p* < 0.05). Thus, Equations (4)–(8) were obtained for natural orange juice and Equations (9)–(13) were obtained for pasteurized apple juice:

y_1_ = 81.27 + 4.57 x_1_^2^ + 5.64 x_3_^2^ (4)

y_2_ = −0.120 − 0.421 x_1_^2^ − 0.379 x_2_^2^ − 0.631 x_3_^2^ (5)

y_3_ = 17.47 − 2.53 x_2_^2^ − 3.96 x_3_^2^ (6)

y_4_ = 11.70 − 4.39 x_1_^2^ − 2.51 x_2_^2^ − 6.04 x_3_^2^ (7)

y_5_ = 17.47 − 2.48 x_2_^2^ − 3.91 x_3_^2^ (8)

y_1_ = 67.61 − 7.68 x_1_ − 1.36 x_3_ + 2.51 x_1_^2^ − 2.40 x_3_^2^ (9)

y_2_ = 3.80 + 1.42 x_1_ + 0.278 x_3_ + 0.148 x_1_x_2_ − 0.215 x_1_x_3_ − 0.551 x_1_^2^ + 0.166 x_3_^2^ (10)

y_3_ = 31.65 + 2.08 x_1_ + 1.00 x_2_ + 1.95 x_3_ + 1.38 x_2_x_3_ + 1.580 x_3_^2^ (11)

y_4_ = 13.60 + 6.43 x_1_ (12)

y_5_ = 31.87 + 2.20 x_1_ + 0.988 x_2_ + 1.96 x_3_ + 1.40 x_2_x_3_ + 1.57 x_3_^2^ (13)

By taking into account the above equations, it can be observed that in apple juice the most significant variables for all dependent colour variables studied were the biosurfactant concentration (x_1_) and the temperature (x_3_), since they are included in most of the formulated equations for the dependent variables (9–13) either as linear or quadratic components; even for the variable a* (y_2_) is significant the interaction between these two variables (Equation 10). However, for the orange juice, the quadratic components of both independent variables (x_1_ and x_3_) were significant for the dependent variables L*(y_1_), a* (y_2_), and ΔE* (y_4_), as can be shown in Equations (4), (5), and (7), respectively. In contrast, for the dependent variables b* (y_3_) and $C_{\mathrm{ab}}^{*}$ (y_5_), the unique significant variables were the quadratic terms of the storage time (x_2_) and temperature (x_3_), according with Equations (6) and (8), respectively. From a statistical perspective, it is possible to confirm that the concentration of the biosurfactant extract (x_1_) is an influential variable in the colour attributes of the two juice matrices studied (pasteurized apple and natural orange), thus affecting the juices’ quality and long-term stability. Moreover, in Table 3 can also be observed the correlation coefficients (r^2^) obtained for all dependent variables (y_1_–y_5_) for both orange and apple juices, with values ranging from 0.87 to 0.96 (Equations (4)–(8)) and between 0.88 and 0.99 (Equations (9)–(13)), respectively. These values suggest that the mathematical model may be accepted for a predictive purpose.

### 3.2. Colour Attributes of Control Juices

The CIELAB colour parameters (L*, a*, b*) were measured in the two fruit juices (pasteurized apple and natural orange) analysed in this work before being included in the experiments of the factorial design. They were considered as control juices to compare the colour changes of the treatments tested under different experimental conditions. In Table 4 are shown the colour parameters of the juice samples employed in this work in comparison with those obtained in other juices that were stabilized by different processing methods (untreated, thermal and non-thermal treatments, sonication, etc.) and even from different cultivars. 

According to Table 4, the colour of the different apple juices is highly influenced by the fruit variety employed to made the juices, although in general all the juices had greater yellowness (b*) than redness (a*) [5,29], which is coherent with the values found in the pasteurized apple juice used in this work (b* value of 34.49 and a* value of 0.90). On the other hand, it was also proved that the stabilization treatment significantly affected the quality parameters of apple juices. The non-thermal methods (usually high-pressure processing) were the more effective procedures to preserve the fruit attributes in these juices [30]. In addition, other researches have also suggested the use of ultrasound treatment as an alternative technology to improve the safety and quality of apple juices by preserving its nutrients and sensory properties [28]. In the case of orange juice, it is well known that its colouring is greatly associated with the carotenoid content [26], which mainly depends on the genotype of each cultivar, climatic and agronomic factors, juice processing, storage conditions, etc. Thus, Fernández-Vázquez et al. [27] have evaluated the colour attributes of untreated juices from five orange varieties and significant differences were found among them regarding L*, a*, and b* parameters, with values in the ranges 56.48–60.66, 12.40–24.60, and 58.12–64.66, respectively, and all of them more oriented to yellowness (b*) than redness (a*). It is important indicate that the natural orange juice employed in this study appears lighter (L* value of 91.75) and less yellow (b* value of 12.48) than the other untreated orange juices previously studied [7,27]. Other authors [3] also confirmed that the colour degradation of orange juices (appearing browning colour) is more evident at elevated temperatures and prolonged storage times, which can be mainly attributed to appreciable changes in the profile of carotenoids through isomerization or oxidation reactions or even caused by other non-enzymatic browning reactions. The influence of the stabilization treatment on the colour parameters of the orange juice was also evaluated by comparing traditional (helical coil heat exchanger, ohmic heating, and mild pasteurization) with other emerging technologies (high-pressure processing). It was proved that high-pressure processing is the best treatment method to ensure the sensory characteristics and quality parameters stay more similar to those of untreated fresh juices [7].

### 3.3. Colour Changes in Natural Orange Juice in the Presence of the Biosurfactant Extract

**Figure 1:**
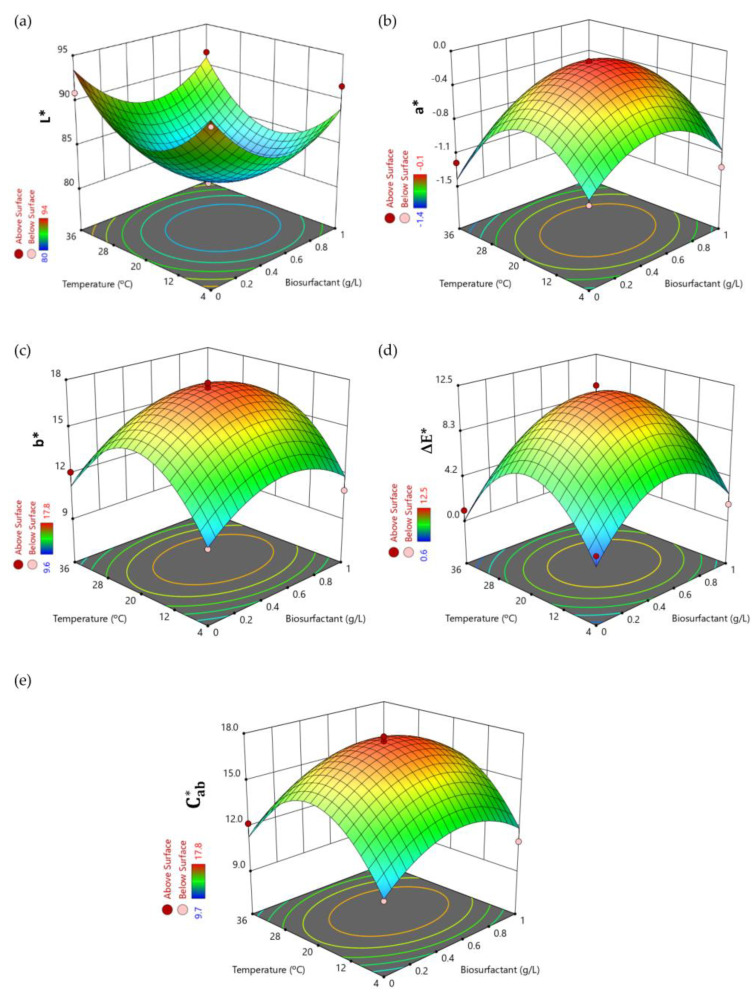
Three-dimensional surface response plots that shown the variation in the dependent colour variables (y_1_–y_5_) in natural orange juice in response to changes in the biosurfactant concentration (g/L) and the temperature (°C) at a fixed storage time of 4 days: (**a**) L*, (**b**) a*, (**c**) b*, (**d**) ΔE*, (**e**) $C_{\mathrm{ab}}^{*}$.

Figure 1 shows surface response plots representing the variation in the five dependent variables studied (y_1_–y_5_) in response to variations in the concentration of the biosurfactant extract and the temperature for a fixed storage time of 4 days; this being the last variable and the least significant in the studied system (Equations (4)–(8)). 

Figure 1a shows the influence of the biosurfactant extract on the lightness (L*), a significant decrease in this variable at biosurfactant concentrations around 0.5 g/L and intermediate temperatures of 20 °C can be observed. For these experimental conditions, L* values decreased to 80.5–82.0 (experiments 13–15 of Table 3), the juices being less bright than in absence of the biosurfactant extract (L* values between 90.9 and 91.9) for the same storage time and different temperatures (experiments 9 and 10 of Table 3). These values (80.5–82.0) are more favourable than others reported in the literature (see Table 4), which compromise lower L* values. This decrease in lightness observed at intermediate experimental conditions (Figure 1a) could be attributed to the possible proliferation of microorganisms causing a slight turbidity in the juice and the release of more acidic compounds, which may be associated with a slight decrease in the pH at intermediate operational conditions. In fact, in a previous work it was proved that there was an increase in biomass growth in natural orange juice up to 0.97–1.07 g/L at temperatures of 20 °C in the presence of 0.5 g/L of CSL biosurfactant extract and a slight decrease in pH from 3.77 to 3.40 [14]. Moreover, lactic acid bacteria, which produce lactic acid that decreases the pH of juice, grow in orange juice at temperatures ranging from 5 to 53 °C; the best conditions being between 30 and 45 °C. This fact it is consistent with the lower L* values observed in Figure 1a. In addition, the Ascomycetes-type fungi also grow under optimal conditions at temperatures ranging from 20 to 30 °C [31]. In Figure 1b,c are shown the variations in the redness (a*) and yellowness (b*), respectively, in response to different concentrations of biosurfactant extract and temperatures. It can be observed that both colour variables reach maximum values at intermediate temperatures (20 °C) and biosurfactant concentrations of around 0.5 g/L. The highest yellowness (b*) values oscillate between 17.1 and 17.8 (experiments 13–15 in Table 3; medium-dose biosurfactant), probably related to the large amount of oxidation carried out by the microorganisms that grow in orange juice. They are followed by experiment 8 (b* value of 16.1; high-dose biosurfactant), and these values significantly decrease to 10.7 (experiment 9 in Table 3) or 12.1 (experiment 10 in Table 3) in absence of the biosurfactant extract for the same storage time and different conservation temperatures (4 °C or 36 °C, respectively). This means that the presence of medium (0.5 g/L) or high doses (1 g/L) of the biosurfactant in orange juice could favour the shift towards the chromatic axis of yellowness (>b*) while decreasing the lightness (<L*). The biosurfactant extract is a brown viscous liquid that can also contribute to increase in the b* values in orange juice; this is in concordance with the results obtained in the present work. The maximum values of the parameter (a*) ranged from −0.11 to −0.13, indicating no orientation towards the redness chromatic axis (negative values of a*) and with a minimum shift towards the greenness, since the a* values are very close to the centre point of the CIELAB colour space.

Figure 1d shows the influence of the biosurfactant extract and temperature in the variable ΔE*, which was calculated with respect to the control juice (before treatment) to analytically quantify the differences found in the juice colour after each treatment tested and to highlight the ability of tasters or consumers to detect these differences. For fruit juices, differences less than 0.5 units in this parameter are not detectable, between 0.5 and 1.5 are slightly noticeable, from 1.5 to 3.0 are noticeable, between 3.0 and 6.0 are well visible, and higher than 6.0 are great [32]. In Table 5 can be observed the visual colour variation of the different trials tested with respect to the control juice solutions, together with the ΔE* values and the yellow/brown component of the colour (A_420_ nm). Regarding orange juices, most of the experiments tested led to browning, in agreement with darker juices (<L*) and with more redness (>a*). However, these differences were not always visually perceived, as can be seen in Table 5 and according to the colour difference threshold (ΔE* value of 2.8 units) previously reported for orange juice consumers [15].

Therefore, the addition of the biosurfactant extract is hardly perceived for treatments 9, 10, 11, and 12 and the colour difference is less than the threshold for treatments 4 and 6. After these six treatments the orange juices showed differences in the L* parameter of less than 2 units, although the juice had a yellowish hue after treatment 6 (b* value of 14.40, higher than the control (12.48)). For the other treatments, different colour shifts were observed. On the one hand, a blanching effect was evident for treatments 3 and 5 (ΔE* = 3.3–3.6), which was verified by a reduction of about −20% in the absorbance value at 420 nm. On the other hand, juice browning occurred for treatments 13, 14, and 15 (ΔE* = 11.1–12.5), with a mean increase of +71% in the absorbance value at 420 nm, followed in decreasing order by treatment 8 (ΔE* = 6.5), with an increased absorbance value at 420 nm of +46%. A higher shift towards brown colour was related to higher yellowness (>b*). This fact is coherent with Figure 1d, where the higher values of colour saturation $\left(C_{\mathrm{ab}}^{*}\right)$ correspond with experiments 13, 14, and 15, with $C_{\mathrm{ab}}^{*}$ values ranging from 17.1 to 17.8, followed by the experiment 8, with a $C_{\mathrm{ab}}^{*}$ value of 16.1 (Table 3). Finally, the orange juices resulting from the treatments 1, 2, and 7 showed a tendency towards greyish hues (ΔE* = 3.2–6.5). It seems that the biosurfactant addition had a negative effect on orange juice colour at a storage temperature of 20 °C, whereas when storing at 4 or 36 °C this effect was suppressed. Therefore, the addition of biosurfactant extract did not affect the colour perception of the juice under refrigeration, these being the conditions recommended to store natural orange juice to prevent oxidation and microbial growth.

The composition and concentration of carotenoids in juices are responsible for yellow-orange-red hues. The higher the concentration of carotenoids, the darker the colour of the orange juice. Previous studies have reported that the total carotenoid concentration is negatively correlated with the L* coordinate but positively correlated with the a* coordinate [26,33]. The distinctive colour of these pigments depends on their chemical structures, the number and arrangement of conjugated double bonds, and the geometrical isomers [34]. Individual carotenoids such as ζ-carotene, lutein, zeaxanthin, and (all-E)-violaxanthin are positively correlated with the b* coordinate [33]. In fact, the oxidation and isomerisation reactions of carotenoids play an important role in the colour changes during orange juice processing and storage [3,34]. This fact it is in consonance with the composition of the biosurfactant extract under evaluation as it contains antioxidants [20].

### 3.4. Colour Changes in Pasteurized Apple Juice in the Presence of the Biosurfactant Extract

**Figure 2:**
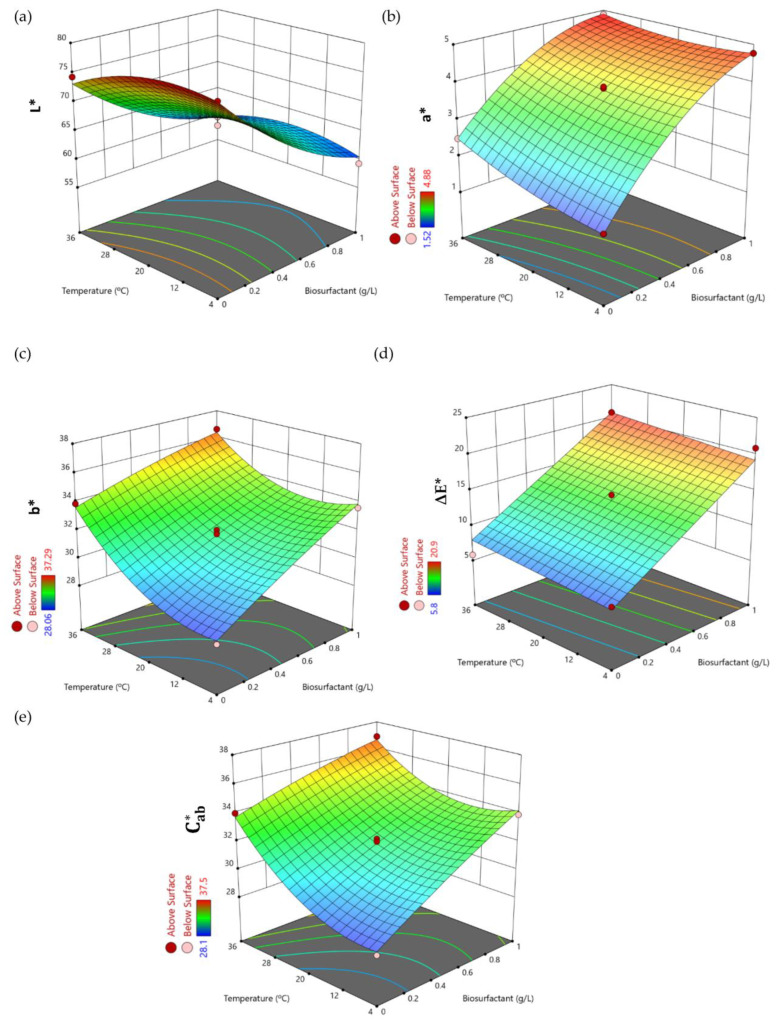
Three-dimensional surface response plots showing the variation in the dependent colour variables (y_1_–y_5_) in pasteurized apple juice in response to the biosurfactant concentration (g/L) and the temperature (°C) at a fixed storage time of 4 days: (**a**) L*, (**b**) a*, (**c**) b*, (**d**) ΔE*, (**e**) $C_{\mathrm{ab}}^{*}$.

Figure 2 shows the surface response plots corresponding to the variation in the five dependent variables studied (y_1_–y_5_) in response to variations in the concentration of the biosurfactant extract and the temperature for a fixed storage time of 4 days; this being the last variable the least significant in the studied system according Equations (9)–(14).

.

Figure 2a shows the influence of the biosurfactant extract on the lightness (L*); a significant decrease in this variable can be observed when the biosurfactant concentration is increased to 1 g/L. In the absence of the biosurfactant extract the L* values ranged from 74.30 to 77.8 (experiments 5, 6, 9, and 10 in Table 3), and these values were decreased to 59.3–63.7 in experiments 3, 4, 7, 8, 11, and 12 in Table 3, corresponding with the presence of biosurfactant at the highest dose or high temperatures (experiments 3, 4). This means that the addition of biosurfactant had a negative effect on the lightness of apple juice, although this effect was less pronounced when the juice was stored at 4 °C. The lower L* values for apple juice containing biosurfactant extract are in consonance with the decrease in polymeric sugar degradation observed in a previous work on presence of biosurfactant extract [14]. In contrast, the other colour parameters evaluated (a*, b*, ΔE*, and $C_{\mathrm{ab}}^{*}$) were increased when the biosurfactant concentration was increased up to the highest dose (1 g/L) at conservation temperatures close to 36 °C (variables b* and $C_{\mathrm{ab}}^{*}$) or even for all temperatures studied (variables a* and ΔE*), as can be observed in Figure 2c,e and Figure 2b,d, respectively. Thus, the highest b* values (between 36.6 and 37.39) were found in treatments 4 and 12 (Table 3), in presence of medium or high concentrations of biosurfactant and always at a high storage temperature (36 °C). The highest colour saturations were also found in the same experiments, with $C_{\mathrm{ab}}^{*}$ values of 36.9 (experiment 12) and 37.5 (experiment 4).

By considering the variable ΔE*, all the treatments carried out on the apple juice caused relevant colour changes that could be visually perceived, as shown in Table 5. A strong browning effect was observed for most of the treatments when compared with the control apple juice, showing values higher than 10 and an increase in the absorbance value at 420 nm that was greater than +20%. This effect was particularly evident, in decreasing order, for treatments 11, 12, 7, 3, 4, and 8, corresponding to the high doses of biosurfactant or medium doses at high storage temperature (ΔE* > 16), followed by treatments 1, 15, 2, 14, and 13, which were associated with the medium doses of biosurfactant at room and low storage temperatures (ΔE* between 11.7 and 14.5). The treatments 9, 6, 5, and 10 showed less total colour difference due to the absence of the biosurfactant extract (ΔE* ranging from 5.78 to 6.77). It is important to highlight that the treatments 5, 6, and 9 led to a decreased browning with respect to control juice, which agrees with a reduction of about −10% in the absorbance value at 420 nm; this is advantageous for the perceived quality (ΔE* values around 6).

For apple juice, polyphenol-mediated browning is a severe problem involving intermediate quinones, which can subsequently undergo secondary condensation reactions synthesizing undesirable brown pigments. Enzymatic and non-enzymatic oxidation reactions can occur, mainly from hydroxycinnamic acids such as chlorogenic and caffeic acids or flavan-3-ols such as catechin and epicatechin. Polyphenol oxidases (PPO) and peroxidases (POD) are responsible for enzymatic oxidation during fruit crushing [35]. These colour changes can also happen during juice storage as a result of non-enzymatic polyphenol oxidation [36]. Furthermore, multiple reaction pathways can be simultaneously involved in non-enzymatic browning. Paravisini and Peterson [37,38] have emphasized the role of reactive carbonyl species (RCS) in Maillard reactions as one of the main browning mechanisms only during long-term orange and apple juice storage (four weeks at 35 °C and ten weeks at 4 °C). This reaction may be favoured by the high concentration of reducing sugars and the low pH values of fruit juices. In this sense, in a previous work it was proved that the addition of a CSL biosurfactant at concentrations higher than 0.4 g/L to a pasteurized apple juice promoted a lower release of total sugars in the juice in comparison with the experiments conducted with lower doses of or no biosurfactant [14]. However, the experiments developed in this work in the absence of the biosurfactant extract (potentially higher sugars) were minorly affected by the browning process, which does not seem to be significantly influenced by the content of sugars. On the contrary, the discoloration may be due to the reduction of o-quinones to colourless diphenols, their reaction with chelating agents to form a colourless adduct, or the irreversible reaction with catechol to hinder the formation of browning complexes [35].

## 4. Conclusions

The results obtained allow us to conclude that the incorporation of the biosurfactant extract into natural orange juice did not affect colour properties when storing the juice at 4 or 36 °C. Only a significant decrease in lightness (<L*) and a visible browning effect (>b* and ΔE* values between 6.53 and 12.5) were observed in the presence of medium or high doses of the biosurfactant extract (0.5 or 1 g/L) combined with intermediate conservation temperatures (20 °C). In the case of apple juice, all of the treatments tested caused relevant colour changes that could be visually perceived by consumers, with ΔE* values higher than 10 in most of cases; although in absence of the biosurfactant extract the ΔE* values were around 6 or higher. Moreover, a strong browning effect was observed for most of the treatments developed when compared with control apple juice. Taking into account the results of this work, it is necessary to carry out sensory panels in order to establish how the biosurfactant extract affects the aftertaste attributes of orange and apple juices. Due to the emulsifier capacity of this biosurfactant extract, juices containing it should probably be sticker; this fact is related to the lower L* values obtained in this work in presence of the biosurfactant extract. In the future it would be interesting to quantify other specific sensory attributes, such as flavour, aroma, appearance, aftertaste, etc., using trained panellists and the quantitative descriptive analysis (QDA) method. The colour parameters quantified in this work could be correlated with those evaluated by the QDA sensory evaluation to establish a relationship between both methodologies associated with the quality of fruit juices, with possible repercussions on the purchase decision making.

## Figures and Tables

**Table 1:** Independent and dependent variables evaluated by the Box–Behnken factorial design.

Independent Variables			
	Units	Range	
Biosurfactant concentration	g/L	0–1	
Storage time	days	1–7	
Temperature	°C	4–36	
Coded independent variables			
	Nomenclature	Equation	Range
Biosurfactant concentration	x_1_	(x_1_ − 0.5)/0.5	(−1,1)
Storage time	x_2_	(x_2_ − 4)/3	(−1,1)
Temperature	x_3_	(x_3_ − 20)/16	(−1,1)
Dependent variables			
	Nomenclature		
L* (lightness)	y_1_		
a* (redness–greenness perception)	y_2_		
b* (yellowness–blueness perception)	y_3_		
ΔE* (total colour differences)	y_4_		
$C_{\mathrm{ab}}^{*}\;$(saturation index)	y_5_		

**Table 2:** Operational conditions considered in this study (expressed as non-coded independent variables) and experimental results obtained for the dependent variables y_1_ to y_5_.

	Non-Coded Independent Variables			Dependent Variables				
Exp	x_1_	x_2_	x_3_	y_1_	y_2_	y_3_	y_4_	y_5_
Orange Juice								
1	0.5	1	4	87.7	−0.76	11.7	4.23	11.8
2	0.5	7	4	89.0	−1.05	10.9	3.21	11.0
3	0.5	1	36	93.4	−1.31	9.60	3.30	9.70
4	0.5	7	36	90.0	−1.40	11.7	1.94	11.8
5	0	1	20	93.9	−1.25	9.60	3.57	9.70
6	0	7	20	90.2	−0.86	14.4	2.58	14.5
7	1	1	20	85.3	−1.05	11.5	6.53	11.5
8	1	7	20	86.4	−0.52	16.1	6.55	16.1
9	0	4	4	91.9	−1.11	10.7	1.87	10.7
10	0	4	36	90.9	−1.23	12.1	0.98	12.2
11	1	4	4	91.5	−1.28	10.9	1.59	11.0
12	1	4	36	91.6	−1.07	12.2	0.60	12.2
13	0.5	4	20	82.0	−0.12	17.5	11.1	17.5
14	0.5	4	20	81.3	−0.11	17.1	11.5	17.1
15	0.5	4	20	80.5	−0.13	17.8	12.5	17.8
Apple Juice								
1	0.5	1	4	66.0	3.68	31.0	14.5	31.3
2	0.5	7	4	67.4	3.50	31.1	13.2	31.3
3	0.5	1	36	62.3	4.17	31.7	18.0	31.9
4	0.5	7	36	63.4	4.15	37.3	17.0	37.5
5	0	1	20	77.1	1.83	28.7	6.45	28.8
6	0	7	20	76.3	1.64	29.1	6.46	29.2
7	1	1	20	61.6	4.38	32.2	18.7	32.5
8	1	7	20	63.7	4.78	34.0	16.6	34.4
9	0	4	4	77.8	1.52	28.1	6.77	28.1
10	0	4	36	74.3	2.49	33.9	5.78	34.0
11	1	4	4	59.3	4.77	33.6	20.9	33.9
12	1	4	36	59.5	4.88	36.6	20.8	36.9
13	0.5	4	20	69.0	3.85	31.2	11.7	31.4
14	0,5	4	20	67.8	3.66	32.0	12.6	32.2
15	0.5	4	20	66.0	3.89	31.7	14.4	32.0

**Table 3:** Regression coefficients and their statistical significance (*p*-value) for the dependent variables (y_1_–y_5_) evaluated in this study as well as the correlation coefficients (r^2^) of the mathematical fit.

Orange Juice										
	**y_1_**	***p*** **-Values**	**y_2_**	***p*** **-Values**	**y_3_**	***p*** **-Values**	**y_4_**	***p*** **-Values**	**y_5_**	***p*** **-Values**
β_o_	81.27	0.0526	−0.120	0.0787	17.47	0.0475 *	11.70	0.0047 *	17.47	0.0483 *
β_1_	−1.51	0.1334	0.0663	0.5225	0.487	0.4107	0.763	0.1556	0.463	0.4306
β_2_	−0.588	0.5177	0.0675	0.5151	1.34	0.0571	−0.425	0.3944	1.34	0.0559
β_3_	0.725	0.4299	−0.101	0.3417	0.175	0.7604	−0.513	0.3124	0.175	0.7589
β_12_	1.200	0.3612	0.0350	0.8076	−0.050	0.9506	0.250	0.7144	−0.050	0.9503
β_13_	0.275	0.8270	0.0825	0.5715	−0.025	0.9753	−0.025	0.9706	−0.075	0.9255
β_23_	−1.175	0.3705	0.0500	0.7288	0.725	0.3887	−0.100	0.8829	0.725	0.3858
β_1_^2^	4.57	0.0144 *	−0.421	0.0312 *	−2.03	0.0517	−4.39	0.0013 *	−2.03	0.0507
β_2_^2^	3.12	0.0540	−0.379	0.0444 *	−2.53	0.0249 *	−2.51	0.0134 *	−2.48	0.0261 *
β_3_^2^	5.64	0.0062 *	−0.631	0.0067 *	−3.96	0.0043 *	−6.04	0.0003 *	−3.91	0.0044 *
r^2^	0.89		0.87		0.90		0.96		0.90	
Apple Juice										
β_o_	67.61	0.0009 *	3.80	<0.0001 *	31.65	0.0016 *	13.6	<0.0001 *	31.87	0.0013 *
β_1_	−7.68	<0.0001 *	1.42	<0.0001 *	2.08	0.0004 *	6.43	<0.0001 *	2.20	0.0002 *
β_2_	0.463	0.4087	0.0012	0.9737	1.00	0.0090 *	−0.550	0.4708	0.988	0.0089 *
β_3_	−1.36	0.0452 *	0.278	0.0006 *	1.95	0.0005 *	0.775	0.3151	1.96	0.0004 *
β_12_	0.727	0.3620	0.148	0.0341 *	0.365	0.3347			0.375	0.3157
β_13_	0.930	0.2561	−0.215	0.0083 *	−0.700	0.0961			−0.725	0.0837
β_23_	−0.0825	0.9139	0.040	0.4682	1.38	0.0100 *			1.40	0.0088 *
β_1_^2^	2.51	0.0209 *	−0.551	0.0001 *	−0.195	0.6074			−0.208	0.5778
β_2_^2^	−0.433	0.5909	−0.0912	0.1461	−0.445	0.2666			−0.433	0.2709
β_3_^2^	−2.40	0.0247 *	0.166	0.0259 *	1.580	0.0068 *			1.57	0.0066 *
r^2^	0.98		0.99		0.98		0.88		0.98	

* *p* < 0.05 significant variables for a confidence interval of 95%.

**Table 4:** CIELAB colour parameters (L*, a*, b*) in untreated and processed orange and apple juices.

Fruit Juice	Treatment	L*	a*	b*	Reference
Orange	Pasteurized	61.42	3.33	53.77	[3]
Orange	Untreated	65–70	4–5	70–75	[7]
	Mild pasteurized	65–70	3–4	70–75	
	High-pressure processing	65–70	2–3	70–75	
Orange	Ultrafrozen	73.41	13.90	68.29	[26]
	Thermal	76.79	8.32	63.38	
Orange (5 varieties)	Untreated	56.48–60.66	12.40–24.60	58.12–64.66	[27]
Orange	Centrifuged	91.75	−1.57	12.48	This study
Apple (6 varieties)	Centrifuged	30.25–62.69	13.1–58.3	20.85–40.91	[5]
Apple	Untreated	19.77	6.75	0.33	[28]
	Ultrasound	19.64	6.35	0.76	
Apple	Untreated	96.77	−0.81	9.69	[29]
Apple (3 varieties)	Untreated	38.9–49.9	0.1–11.1	26.8–36.7	[30]
	Thermal	40.9–59.4	−7.0–4.4	34.3–43.0	
	High-pressure processing	37.6–51.2	−2.9–6.3	32.0–39.1	
Apple	Pasteurized/centrifuged	79.83	0.90	34.49	This study

**Table 5:** Visual colour variation of the different experiments corresponding to the Box–Behnken factorial design with respect to the control orange and apple juices. The ΔE* values versus control solutions as well as the absorbance values at 420 nm (A_420_) are also included.

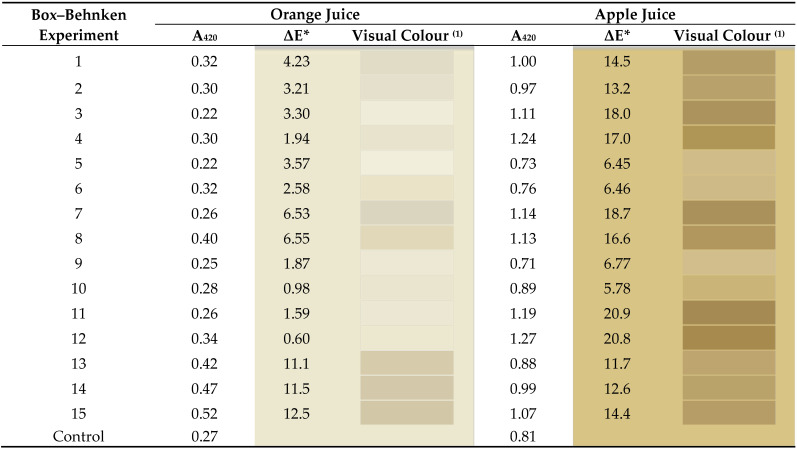

^(1)^ Each colour was obtained by spectrophotometric measurements in CIELAB coordinates (L*, a*, b*) that were converted into RGB values.

## Data Availability

Data is contained within the article.
